# Prediction of pest pressure on corn root nodes: the POPP-Corn model

**DOI:** 10.1007/s10340-016-0788-x

**Published:** 2016-06-21

**Authors:** Annika Agatz, Roman Ashauer, Paul Sweeney, Colin D. Brown

**Affiliations:** 1 Environment Department, University of York, Heslington, York, UK; 2 Syngenta, Jealott’s Hill, Bracknell, UK

**Keywords:** Population model, Integrated pest management, *Diabrotica*, Node injury, *Zea mays*, Pest

## Abstract

A model for the corn rootworm *Diabrotica* spp. combined with a temporally explicit model for development of corn roots across the soil profile was developed to link pest ecology, root damage and yield loss. Development of the model focused on simulating root damage from rootworm feeding in accordance with observations in the field to allow the virtual testing of efficacy from management interventions in the future. We present the model and demonstrate its applicability for simulating root damage by comparison between observed and simulated pest development and root damage (assessed according to the node injury scale from 0 to 3) for field studies from the literature conducted in Urbana, Illinois (US), between 1991 and 2014. The model simulated the first appearance of larvae and adults to within a week of that observed in 88 and 71 % of all years, respectively, and in all cases to within 2 weeks of the first sightings recorded for central Illinois. Furthermore, in 73 % of all years simulated root damage differed by <0.5 node injury scale points compared to the observations made in the field between 2005 and 2014 even though accurate information for initial pest pressure (i.e. number of eggs in the soil) was not measured at the sites or available from nearby locations. This is, to our knowledge, the first time that pest ecology, root damage and yield loss have been successfully interlinked to produce a virtual field. There are potential applications in investigating efficacy of different pest control measures and strategies.

**Electronic supplementary material:**

The online version of this article (doi:10.1007/s10340-016-0788-x) contains supplementary material, which is available to authorized users.

## Key message

- The corn rootworm is a pest of international importance in corn production due to larvae feeding on roots.
- Deciding on the necessity for and optimal decision on type of control measure is challenging because damage and yield loss vary with region and season.
- A model capable of predicting root damage specific to region and season has been developed and provides a powerful tool to support decision-making processes for both provider and user of pest control measures.

## Introduction

The corn rootworm *Diabrotica* spp. (Coleoptera: Chrysomelidae) is a univoltine pest in corn [*Zea mays* subsp. *Mays* (*L.*)] production which has become a pest of international importance due to its presence in the USA, Canada, Mexico and Europe (Meinke et al. 2009). Yield loss and expenses associated with rootworm damage exceed costs of $1 billion per year in the USA alone (Tinsley et al. 2015). Adult beetles lay eggs from the middle of the summer to late autumn into the upper 30 cm of soil (Vidal et al. 2005). Eggs overwinter and develop to new adults below ground over the summer to then emerge from the soil, mate and lay eggs for the next growing season. The larvae, which hatch from the eggs around early summer, pass through three instar phases before pupation and emergence as adult beetles (Meinke et al. 2009). All larval instars depend on foraging on corn roots for successful development; this can cause substantial damage which is manifested in reduced growth and lodging of corn plants (Meinke et al. 2009). The damage caused depends on a variety of factors including, but not limited to, pest pressure, planting time, row spacing at planting, climate and soil type.

Damage control strategies for corn rootworm include the use of crop rotation, the use of Bt corn and the application of plant protection products against adults as foliar application and/or against larvae as seed treatment or soil application (Hodgson 2008). Both the need for damage control measures and the effectiveness of those measures in controlling pest populations within economic thresholds will vary in space and time. This presents challenges to farmers in deciding on the necessity for and optimal decision on type of control measure, and to assessors and developers of control measures to judge the overall (region- and season-independent) efficacy of a control measure. The development of a temporally and spatially explicit population model which supports the prediction of root damage and yield loss that is specific to region and season could thus be a vital step towards the provision of a powerful tool to support decision-making processes for both provider and user of pest control measures.

There has been extensive research on simulation of corn rootworm including the development of modelling tools to predict temporal egg hatch and adult emergence (Nowatzki et al. 2002; Schaafsma et al. 1991; Stevenson et al. 2008), the development of cohort population models (Elliott and Hein 1991; Elliott et al. 1990; Mitchell and Riedell 2001; Mooney and Turpin 1976) and the development of models simulating the spatial distribution of adult beetles across fields, between fields and at even larger scales (Hemerik et al. 2004; Knapic et al. 2009; O’Rourke et al. 2011; Onstad et al. 1999; Stevenson et al. 2008). Models were developed to investigate pest management strategies in terms of the use of crop rotation (Szalai et al. 2014) or a combination of crop rotation and pesticide application (Krügener et al. 2011). Additionally, linear models are available that can convert root damage into yield loss (Dun et al. 2010; Tinsley et al. 2013). Despite this work, we are not aware of any model that has been developed to simulate directly root damage and thus to link explicitly pest ecology, root damage and yield loss.

The aim of this research was the development and evaluation of a population model that combines the temporally explicit and spatially specific appearance of the different life stages of the corn rootworm in the soil profile with simulation of population abundance and damage to corn roots. The model is developed mainly using information originating from research on the non-rotation-resistant variant of the western corn rootworm *Diabrotica virgifera virgifera* LeConte, but may be adaptable to other species in the rootworm complex. The POPP-Corn model uses the node injury scale for root damage assessment (Oleson et al. 2005) as its main output to allow direct comparison with observations of damage in the field.

## Methods

### Model overview

The POPP-Corn model is an annual spatially and temporally explicit and temperature-dependent individual-based model (IBM) for all life stages of the western corn rootworm, *Diabrotica virgifera virgifera*; the model is coded in NetLogo 5.0.5 (Wilensky 1999). POPP-Corn predicts the development of rootworms in corn with a particular focus on the temporally explicit positioning of individuals and roots in a two-dimensional soil profile and an explicit link between pest ecology and root damage/yield loss. The individual-based population model simulates the development of eggs, the three larval instars (L1, L2 and L3) and the pupa of the corn rootworm, where transition of individuals between life stages is controlled by temperature-dependent developmental rate functions. The model includes a spatially and temporally explicit sub-model for development of corn roots which are assumed to be the only food source for all larval stages of the rootworm. Individuality of the pest does not derive from stochasticity within individual characteristics such as developmental rate, feeding rate or movement (as commonly conducted in IBMs), but rather from the spatially variable environment for individuals (i.e. food availability and temperature). The POPP-Corn model is spatially explicit in itself because the appearance of larvae and roots is modelled in a two-dimensional soil profile; this is necessary to predict accurately how root feeding varies with soil type because larval movement (and thus foraging success and survival of both larvae and roots) depends on the soil type. Region-specific weather data (temperature and precipitation) are driving variables within the model. These weather data are used to generate two-dimensional temperature and water content profiles for the soil layers modelled which in turn drive the dynamics between pest and root system.

**Fig. 1:**
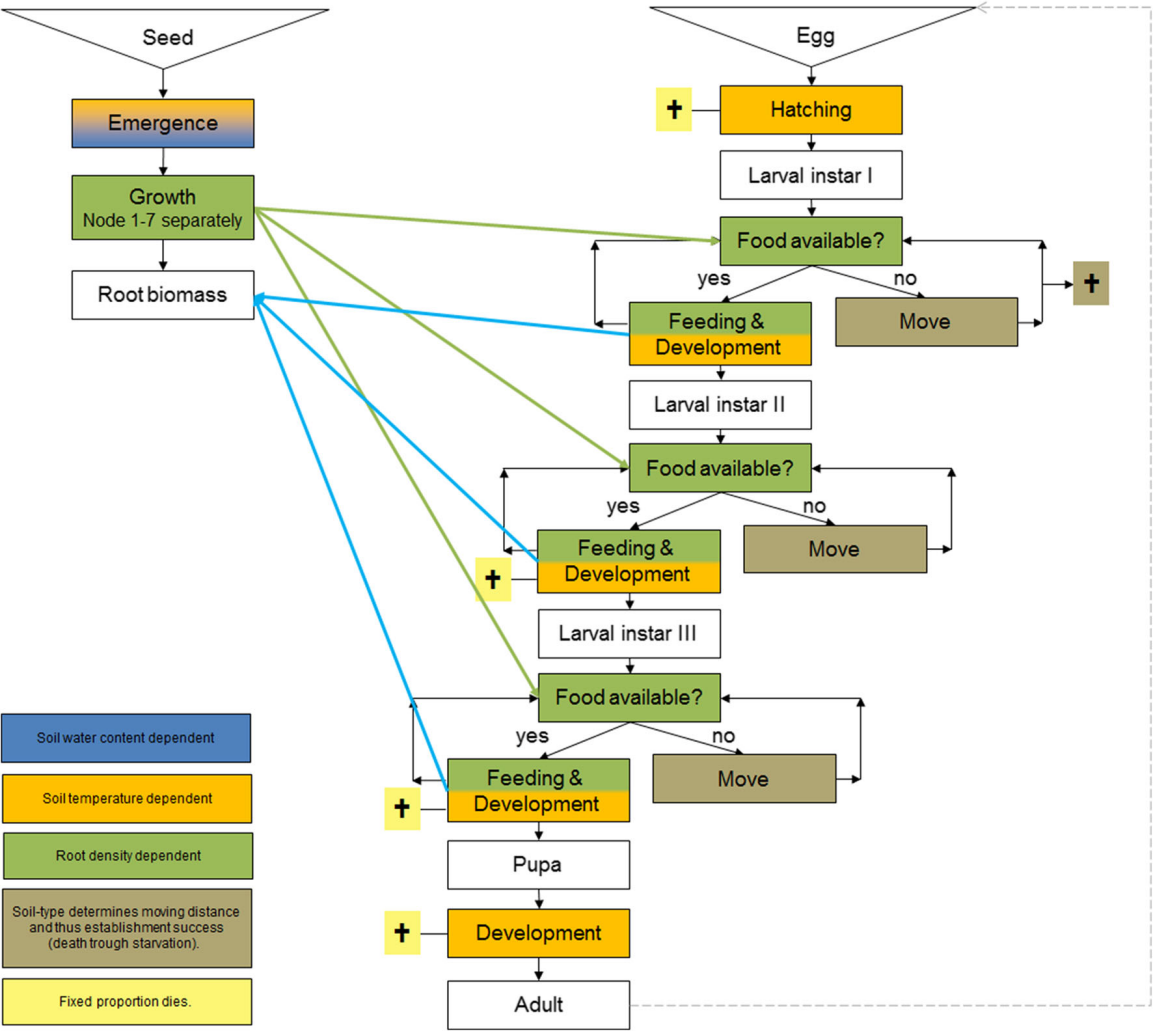
Schematic model description of the POPP-Corn model

POPP-Corn has been developed to simulate the root system of one representative plant in a field with a row spacing of 76 cm in a two-dimensional profile. POPP-Corn represents a vertical soil profile of 76 × 100 cm (*x*- and *y*-axes) and 1 cm depth (*z*-axis). Each patch/grid represents 1 cm^3^. Most processes are updated once per hour, and none of the processes is updated less frequently than once per day. The model runs from the beginning of the calendar year through to the day of node injury assessment using Julian days as temporal measure. A schematic overview for the POPP-Corn model is presented in Fig. 1.

### Input data requirements

A site-specific temperature profile (in °C) is needed for the POPP-Corn model, supplied as hourly temperatures (starting on Julian day 1) throughout the soil profile in 1 cm increments. It is expected that such profiles will normally be generated from weather data using an independent mathematical model (e.g. MACRO; Larsbo et al. 2005). Additionally, hourly water content for the patch/grid where the seed is located (here fixed to the middle of the row at a depth of 5 cm) is needed for a period of 2–3 weeks starting on the sowing day. Further requirements for the model set-up are the pest pressure (i.e. number of eggs), the baseline temperature (in °C) for egg hatch in the region for which the model is set-up, the planting date (in Julian days), the time from planting (in days) at which damage assessment should take place, the soil type and the specification on whether tillage or no-tillage is assumed for egg distribution in the soil.

### Model design

Overall, the POPP-Corn model consists of seven main procedures (root growth, oviposition, pest development, larval movement, pest survival, larval feeding and damage assessment) which use a total of 26 parameters across all processes excluding spatial root simulation (Table OR1), 14 variables and five parameters for spatially explicit root simulation (Table OR2) and several additional variables across all procedures (Table OR3).

Table 1 summarises the underlying POPP-Corn model assumptions grouped according to the main processes discussed within the corresponding sections in the model description.

**Table 1:** Assumptions within the POPP-Corn model

Model process	Assumption
Root growth	Root emergence depends on temperature and water content at sowing depth whereas root development in terms of appearance of new root segments and root mass only depends on the average soil temperature and is independent of water content
	The only process causing root density decline is active larval feeding and root mortality caused by pruning
	Root segments are not directly connected to another
	Root growth along the *z*-axis does not differ with distance from the seedling
Oviposition	Sowing of the crop without additional tillage does not change the egg distribution in the soil
	Egg distribution in the soil is normally distributed from the centre of the furrow towards the row when no-tillage occurs
	There is uniformity in the position of row and furrow within the field between seasons
Larval movement	Larvae only move when they forage. There is no movement when root density at their location is sufficient to supply their feeding
Pest development	Male and female immature stages of the corn rootworm develop at the same rate up to pupation
	Developmental rates for all life stages are definite, and variations in development derive only from differences in temperature and food availability throughout the soil profile
	Developmental baseline temperatures for all life stages, except for eggs, are constant and do not vary with location, as has been observed for egg development
Pest survival	Mortality for 1st instar larvae only depends on foraging success and has no time-dependent component (in contrast to all other life stages)
Larval feeding	Larvae within one patch share the local food available equally if not enough food is available to fully satisfy the hunger of all

#### Root growth

The root development sub-model is temporally and spatially explicit and specific for corn root nodes 1–7. Root development occurs in two parts. The first part is the time-dependent, spatially restricted (but within these boundaries stochastic) appearance of new root segments. When a node has been developed, between null and three new root segments are created randomly every day for each node. Root segments are assigned to one of the nodes of the root system but are not interlinked to each other (no actual network is produced) because in a two-dimensional profile (*x*- and *y*-axes) with a 1-cm depth (*z*-axis) root segments that appear cannot be assumed to be directly connected to another in contrast to connections in reality that span across the *z*-axis and build a root network. This part of the root development sub-model is dependent on temperature and water content in accordance with the details given in the Illinois Agronomy Handbook (2009) and the CORN Newsletter (Lindsey and Thomison 2012). Root growth is restricted to average soil temperatures [ST_a_] between 10 and 30 °C. Root emergence only occurs when the temperature at sowing depth (fixed to 5 cm below the soil surface) is >10 °C (baseline temperature), when heat accumulation calculated with a baseline temperature of 10 °C reaches 43 °C (Lindsey and Thomison 2012) and when the water content at sowing depth is >30 % (Illinois Agronomy Handbook 2009). Root expansion in space (i.e. the development of new root segments) is limited to the first 56 days after root emergence for each node as this duration coincides with observations from Peng et al. (2012) where after roughly 80 days the total root mass decreased. Decrease in root mass has not been implemented into the model as natural root decline is assumed to have no impact on root mass until root damage caused by larval feeding has completed and thus has no impact on the determination of node injury. Root expansion in space is root node specific (i.e. node 1 develops in a different place than node 2, etc.) but their actual positioning in space is randomised within horizontal and vertical boundary conditions. These boundary conditions are characterised by the horizontal random root distribution [hrrd(1–7)] which determines the distance of the root segment from the horizontal centre of the soil profile and the vertical random root distribution factor [*vrrdf*(*1*–*7*)] which determines the distance of the root segment from the soil surface downwards. The latter is a factor because the actual vertical positioning of the root segment [vrrd(1–7)] is given by multiplication with the age of the corresponding root node [ageN(1–7)]. The appearance of a root node in time [*N*(1–7)d] occurs at a set number of days after root emergence, from which point onwards the number of root segments and their positioning is preset in a randomised design.

Development of root mass [output in total root mass per 7.6 L of soil volume (derived from the model dimension)] is assumed to be linear (Clark et al. 2006) and described via a root mass increase per root segment [*rg*]. The calculation of root mass [Rm] resulting from the root mass increase within each patch (Rd_i_) constitutes the second part of the root growth sub-model. Growth of each root segment is limited to 30 days after appearance of this particular segment (Rd_max_).

Growth of root segments is influenced by the pest pressure from local larval feeding on roots [Lf_i_] determined by the local number of larvae per larval instar [Nl1_i_, Nl2_i_ and Nl3_i_] and the feeding rates of the three larval instars [*frl1, frl2* and *frl3*]. If root segments are damaged, growth is limited due to their loss of function, and when root segments are fully eaten and pruned other root segments are pruned (Gavloski et al. 1992). These processes are implemented into the POPP-Corn model via a sub-process called pruning [PRU]. Pruning is incorporated with one parameter [*pru*] that influences the reduction in root growth and thus influences total root mass, and is used in combination with the number of dead root segments per day [Ndr] to determine the probability of remaining root segments dying during the following day as a result of larval feeding. More detailed information on the incorporation of pruning is given in the section on larval feeding.

#### Oviposition

This procedure in the POPP-Corn model handles the spatially explicit positioning of eggs in the autumn preceding the calendar year in which the model is running. The autumn count of eggs [Ne] is used because natural egg mortality during winter is separately implemented in the model (see section “Pest survival” for details).

Positioning of eggs is generally randomised within two boundary conditions and can be simulated for tillage or no-tillage. When tillage (conventional) is selected, the eggs are homogeneously distributed in the horizontal plane and vertical distribution is as given by Vidal et al. (2005) (21 % of eggs within the upper 10 cm [*ed1*], 45 % of eggs in 10–20 cm [*ed2*] and 34 % of eggs in 20–30 cm [*ed3*] of the soil). When no-tillage is chosen, the vertical distribution remains according to Vidal et al. (2005) with the additional limitation of no eggs being in the upper 2 cm (Kirk 1979), but eggs are additionally horizontally concentrated towards the furrow following a random normal distribution [combination of various observations (Kirk 1979, 1981a, b; Pruess et al. 1968)] expressed with the horizontal egg normal distribution [Hend]. Horizontal placement of eggs is not limited by the left and right edge of the soil profile. Hence, if Hend determines placement of eggs outside the soil profile on the left-hand side, eggs are placed on the right-hand side of the soil profile and vice versa. This boundary condition, which also applies to larval movement, allows the single root system modelled to represent the average behaviour of a uniform corn field.

Oviposition in the model assumes that row and plant positioning in the same field is identical in successive years and that the number of eggs per plant is uniform across the field. This assumption has been made despite the knowledge that even in a uniform corn field larval appearance is not uniform and that row and plant positioning plays a vital role for larval appearance (Toepfer et al. 2007) and thus egg numbers per plant. Conventional tillage distributes eggs to near uniformity between row and furrow (Pruess et al. 1968) and thus makes the assumption valid under tillage. Under no-tillage conditions, information on the exact placement of plants within the field from both years (i.e. that simulated and the preceding one) would be necessary, but is not available.

#### Pest development

The development of all soil-based life stages (eggs, larval instars 1–3 and pupa) depends on the local soil temperature [ST_i_] considering a baseline temperature for development that differs for each life stage (Table OR4). Development of any life stage only occurs when the local temperature is greater than the respective baseline temperature. Information in the literature suggests that the baseline temperature for egg development is region specific (Davis et al. 1996), so this baseline temperature [Bte] is a variable within the POPP-Corn model (input parameter). For all other life stages, such information is not available in the literature to our knowledge. Thus, the baseline temperatures for all larval stages and for pupa [Btl1, Btl2, Btl3 and Btp] are fixed to the values calculated from developmental times measured at various temperatures given in the literature (Jackson and Elliott 1988).

For all larval stages and pupa, the relative development [DevL1, DevL2, DevL3, DevP] is tracked on a scale of zero to one, at which point the individual enters the next developmental phase. Development is updated hourly so that movement of individuals does not necessitate interpolation of hourly temperature changes. The development for all life stages, except eggs, is linearly dependent on the local soil temperature [ST_i_] to a maximum at 30 °C beyond which any further increase in temperature does not result in an increase in the developmental rate. Egg development [DevE] is described similarly with the difference that the developmental rate beyond the temperature of maximum development decreases rather than stagnates (Schaafsma et al. 1991). The development of each life stage is described by one parameter, respectively [*de, dl1, dl2, dl3* and *dp*], which describes the relative development per hour and °C above the surrogate baseline temperature calculated from published data (Jackson and Elliott 1988; Levine et al. 1992; Schaafsma et al. 1991; Wilde et al. 1972; Wilstermann and Vidal 2013).

A local starvation variable [Starv_i_] is included into the POPP-Corn model to account for intraspecific competition for food when locally available root mass is not sufficient to feed all larvae within the patch at a given time. This variable influences individual development of larval instars 1–3 by reducing development within the starvation period due to reduced feeding. The starvation variable is defined as the ratio between the number of root segments [Nrs] and the number of larvae [Nl] within the patch [i].

#### Larval movement

Larval movement within the POPP-Corn model is a specific event (moving one patch forward) that happens every hour when triggered and that is influenced by soil type and food availability. The soil type determines the frequency of movement whilst food availability determines the direction of movement. Dependency on soil type has been incorporated for the three soil types tested by Strnad and Dunn (1990), namely sand, sandy loam and silt loam. Larvae can move every hour in sand [*mf*_sand_], every 4 h in sandy loam [*mf*_sandy_] and every 6 h in silt loam [*mf*_silt_]. Movement can be to directly adjacent patches or diagonally resulting in daily movement ranges of 24–33.9 cm in sand, 6–8.5 cm in sandy loam, and 4–5.7 cm in silt loam. Furthermore, movement is restricted to happen only when the food in the patch is not sufficient to feed all larvae within that patch. When local available root segments are fewer than the number of larvae within that patch [Starv_i_ < 1], the model triggers reduced feeding of larvae and the urge for larvae to forage for alternative food (i.e. movement). Individuals start starving and move towards the patch within “sensing distance” [*sd*] (set to be 5 patches) that has the highest root mass. Larvae that are located outside “sensing distance” (more than 5 patches away from any root) move randomly forward at a rate of one patch per moving event. As larvae at different instar are known to feed preferentially on roots of differing diameter/age, movement of all three instars is directed towards the preferred root type rather than towards all roots in general. Larval instar 1 move towards the patch with the largest mass of roots <0.00094 g/patch [*l1m*], larval instar 2 move to the patch with the largest mass of roots between 0.00095 and 0.002 g/patch, and larval instar 3 move to the patch with the largest mass of roots between 0.002 [*l2m*] and 0.005 g/patch [*l3m*] (approximation following Clark et al. 2006). Roots of higher mass (>0.005 g/patch) are assumed to be too old (woody) to be attractive to the larvae because they are harder to eat and less likely to exude CO_2_ (which is the molecule attracting the larvae to the roots) (Strnad and Bergman 1987a, b; Strnad and Dunn 1990).

Horizontal movement is not limited by the left and right edge of the soil profile. Hence, if the larva moves outside the soil profile on the left-hand side, it reappears on the right-hand side of the profile and vice versa. This boundary condition, which also applies to egg placement, allows the single root system modelled to represent the average behaviour of a uniform corn field.

#### Pest survival

Natural mortality within the IBM occurs in three different ways depending on which life stage is considered. Egg mortality [*me*] is the only fixed but normally distributed parameter to account for the observation that a fixed percentage of all eggs laid in the last autumn does not hatch (i.e. does not become a larva) in spring. Strictly, this parameter describes not only egg mortality, but also includes the relatively small percentage of eggs which remain dormant for more than one winter (Meinke et al. 2009).

The relative mortality of larval instars 2 [*ml2*] and larval instars 3 and pupa together [*ml3*] is represented in the model according to the average and standard deviations observed by Toepfer and Kuhlmann (2006). In contrast to egg mortality, mortalities for larval instar 2 and 3 [Sl2, Sl3] are time dependent as instantaneous mortality of the larvae after reaching this life stage would unrealistically eliminate individuals without their temporal contribution to root damage via feeding. The temporal aspect of mortality is incorporated taking into account the developmental rate of individuals [DevL2, DevL3] rather than time itself as this approach allows the incorporation of temperature dependency of mortality because development is temperature dependent. All mortalities incorporated into the model derive from life-tables constructed from observations in the field in Hungary (2000–2002) by Toepfer and Kuhlmann (2006). A rational explanation for the causes of the observed mortality is not available for these life stages. More information for the cause of observed mortality was available in the literature for larval instar 1. A correlation between success of larval establishment on roots and larval survival provides evidence that survival depends on the success of finding food and thus starvation (Strnad and Bergman 1987a, b). The data from Strnad and Bergmann (1987b) on larval establishment success (i.e. foraging and staying at a place where food is available and feeding on this food) as a function of time of starvation were fitted with a Sigmoidal Hill function with three parameters [*ml1a*, *ml1b*, *ml1c*] in SigmaPlot (version 12.5, Systat Software, San Jose, CA). The resulting function served as reverse interpretation of larval instar 1 survival because when larvae do not establish they will consequently die. We decided to use establishment success [Estab] over starvation time [L1a] as a proxy for survival, rather than the survival data *per se.* If an instar-1 larva does not establish at a root, then it cannot contribute to root damage and this becomes important for accurate simulation of root loss. Establishment success declines faster with starvation time than actual survival (Strnad and Bergman 1987a, b). Thus, if survival would be simulated rather than establishment, there would be a chance of root feeding from instar 1 larvae which actually does not occur in the field.

#### Larval feeding

Larval feeding [TLf] in the IBM is described with four parameters [*frl1*, *frl2*, *frl3* and *pru*] and does not only consist of direct feeding of all three larval instars [Dlf1, Dlf2 and Dlf3] but also describes the root pruning caused by active feeding [PRU]. It was observed that between 26 and 38 % of all roots can be pruned by feeding of rootworm larvae (Gavloski et al. 1992), and this was assumed to play a vital role for prediction of rootworm damage as the relative mass of roots pruned can exceed the root mass that is eaten.

Data obtained by Clark et al. (2006) can be recalculated to an average feeding rate of 0.000014 g/larva per hour. It was, however, not possible to use the published information to parameterise the feeding rate of the three different larval instars. Separate feeding assays with the three larval stages conducted with *Diabrotica balteata* as representative alternative species and undertaken over 24 h generated a similar feeding rate for instar 1 [*frl1* = 0.000015 g/larva per hour]. The feeding rate for larval instars 2 [*frl2*] and 3 [*frl3*] roughly doubled with each instar. Details on the feeding assays can be found in the Online Resource for this manuscript. These feeding rates describe the root mass directly eaten by the larvae, but ignore the secondary loss of root mass from pruning. Thus, the implementation of a pruning parameter [*pru*] was vital for accurate damage assessment. The pruning parameter describes the indirect effect on root mass via a reduction in the probability of root segments surviving the next day, with a fixed percentage of every root segment being lost due to larval feeding. The current sub-model for root development (where root segments are not building an actual root network) did not allow spatially explicit simulation of the mortality of root segments as it appears in the field. A randomised survival of remaining root segments was taken to be the most appropriate integration of pruning. Larval feeding and pruning result in a reduction in root growth caused by reduced ability for uptake of water and nutrients by the root system. We decided to implement reduced root growth as being directly linked to induced root mortality using the same parameter [*pru*] because root growth in our model is not limited by water and/or nutrient uptake; thus, even if information was available on the impact of larval feeding on water and nutrient uptake, there is currently no process in the model to account for such a relationship.

#### Damage assessment

The node injury scale (NIS) used for damage assessment on roots in the field is recorded on a scale from 0.00 to 3.00. The number before the dot indicates how many full node equivalents are lost. Zero means no root loss, and 3.00 means three or more node equivalents are lost (Oleson et al. 2005). The numbers after the dot represent the percentage of a full node lost. So a NIS of 1.36, for example, means that one full node (or its equivalent over all nodes) plus an additional 36 % of another node (or its equivalent over all nodes) is lost due to larval feeding.

The root growth sub-model runs twice in parallel (identical runs) at each model run, once pest free and once with pest present to allow assessment of root damage against root growth in the absence of pest pressure. As root growth is modelled to be node specific (node 1–7), it is known at any given point how much of any node has been eaten and pruned. By adding the percentage of roots lost from every node, a total of lost roots expressed as one-node equivalents can be determined. Every value >300 % has an assigned NIS of 3.00 because more than three one-node equivalents are lost. Values lower than 300 % get an assigned NIS equivalent to the total of lost roots as one-node equivalents divided by 100.

Root damage assessed as the node injury value can be transformed into a direct measure of yield loss using the correlations between these two measures reported by Dun et al. (2010) and Tinsley et al. (2013). A one unit difference in the node injury scale equates to a yield loss of 16.5 ± 1.9 % on average in the US Corn Belt.

### Data used for model calibration and model evaluation

The two-dimensional representation of a much more complex three-dimensional root model for corn (Pagès et al. 1989) and the root distribution predicted by a two-dimensional soil plant system model (Hansen et al. 1993) were used to calibrate the appearance and distribution of root segments within the POPP-Corn model. The developmental rate for growth of each root segment was calibrated by fitting modelled root mass to observations made by Anderson (1988).

Four sets of field data were used to create model input files, to perform a model calibration and to undertake the model evaluation. As the model is designed to be region and season specific, all data used were spatially and temporally explicit. Here, we present data originating from central Illinois, USA for years between 1991 and 2014, focusing on a region close to Urbana, Illinois, USA.

Weather data from Bondville, Central Illinois, USA (Water and Atmospheric Resources Monitoring Program, Illinois Climate Network 2015) were used to generate temperature and water content profiles for the years 1991–2014 using the soil hydrological model MACRO (Larsbo et al. 2005). The input for this model is summarised in the Online Resource. Generated outputs were used for all POPP-Corn simulations.

The literature provided information on when rootworm larvae appeared for the first time each season (Krupke and Bledsoe 2014) over the last 24 years and when adult beetles were first observed in central Illinois over the last 14 years (Pest & Crop Newsletter (2014(13), 2013(14), 2012(12), 2010(13), 2009(15), 2008(14), 2007(15), 2006(14), 2005(15), 2004(15), 2003 (16), 2002(16), 2001(14), 2000(16)). These data (given in Table OR5) were used to compare simulated pest population development with field observations without calibration of the model.

A field study was undertaken in 2014 in Monticello, Illinois, USA, to test the efficacy of different control measures to suppress rootworm infestation (unpublished data, Syngenta). In preparation for this efficacy study, the soil characteristics were measured and the pest pressure (measured as the number of rootworm eggs in the soil) was determined in the preceding autumn. The efficacy trial included an area of the field that served as untreated control. The NIS from this control plot was used to calibrate pruning within the POPP-Corn model. Information on soil characteristics is detailed in the Online Resource and has been used for the simulation of the environmental input data to generate temperature and water content profiles representative for this soil type. The observed egg density of 137 eggs/7.6 L of soil served as a representative pest pressure for corn fields in central Illinois characterised as suitable for studies on corn rootworm control/management because pest pressure is maximised through the planting of capture crops in the preceding year.

We could not find further field studies where natural pest pressure, measured as abundance of eggs in the soil, had been recorded in the same field as root damage/yield loss. Thus, we were not able to evaluate the root injury prediction from our model further by direct input of field-specific natural pest pressure. There were, however, control plots of another ten efficacy trials conducted in Urbana, Illinois, in the years 2005–2014 published in the Annual Summary of Field Crop Insect Management Trials “On Target” from the University of Illinois Extension and Department of Crop Sciences. Results of these trials (presented in Table OR5) were used to evaluate simulations of root damage against field observations; the simulations were undertaken assuming that the pest pressure observed in Monticello in 2014 was representative of pest pressure for all studies. This is a relatively large assumption, but it should be noted that all studies included a capture crop in the preceding season to encourage laying of eggs by adult rootworms, thus ensuring intermediate to high pest pressure during the following year.

The baseline temperature for egg development was set at 11 °C for all POPP-Corn simulations because this threshold temperature has been accepted by most authors (Schaafsma et al. 1991); it should be noted that calibrated baseline temperatures reported in the literature for best fit prediction of egg hatch range between 10.8 to 13.8 °C (Wilde et al. 1972; Jackson and Elliott 1988; Schaafsma et al. 1991; Levine et al. 1992; Wilstermann and Vidal 2013). All simulations were repeated 40 times, and average values of these repetitions are presented in all results.

## Results

### Model calibration

Only the parameters for the root growth sub-model dealing with the spatial appearance of roots segments and the pruning parameter were calibrated. Apart from the feeding rates for the three larval instars which were experimentally determined (see Online Resource for detail), all other parameters derive directly from the literature or were calculated from literature data without additional calibration. Calibration of the root growth sub-model was conducted by visually comparing root appearance in time and space with outcomes of two other root developmental models (Figure OR1). It was tested whether the current model gives a good simulation of corn root mass development by comparing outcomes with empirical data and the simulations of another root developmental model (Figures OR2). Overall, the current model gives a good simulation of corn root development within the period of active larval feeding.

After visually fitting the pruning parameter by comparing the observed node injury from the control plot of the field study in Monticello 2014 with outcomes of simulations over a range of values for pruning (Figure OR3), we simulated this field study again and recorded the relative reduction of root mass caused by pruning. Pruning, excluding the active feeding by larvae was simulated to be 28.5 % (95 % CI 19.7–37.3 %) across the whole season, which is comparable to observations of average pruning between 26 and 38 % found in the literature (Gavloski et al. 1992). This is an indication that the connection between the root growth sub-model and the rootworm feeding procedure is implemented in a reasonable fashion.

### Model evaluation

Across the 10-year data set of field studies, planting time, the day of damage assessment after planting, the observation of first larval appearance and first adult appearance and the NIS observed in the control plots in Urbana, Illinois, USA, varied strongly with season. All temporal aspects varied by roughly 1 month (planting time: 32 days, damage assessment: 34 days, larval appearance: 37 days and adult appearance: 26 days), and the NIS ranged from 1.25 to 2.77. Similar variation over the years was observed in the simulations. For example, the difference across seasons in first larval appearance and first adult appearance was simulated to be 36 and 29 days, respectively, whilst the NIS was simulated to range between 0.82 and 2.95.

**Fig. 2:**
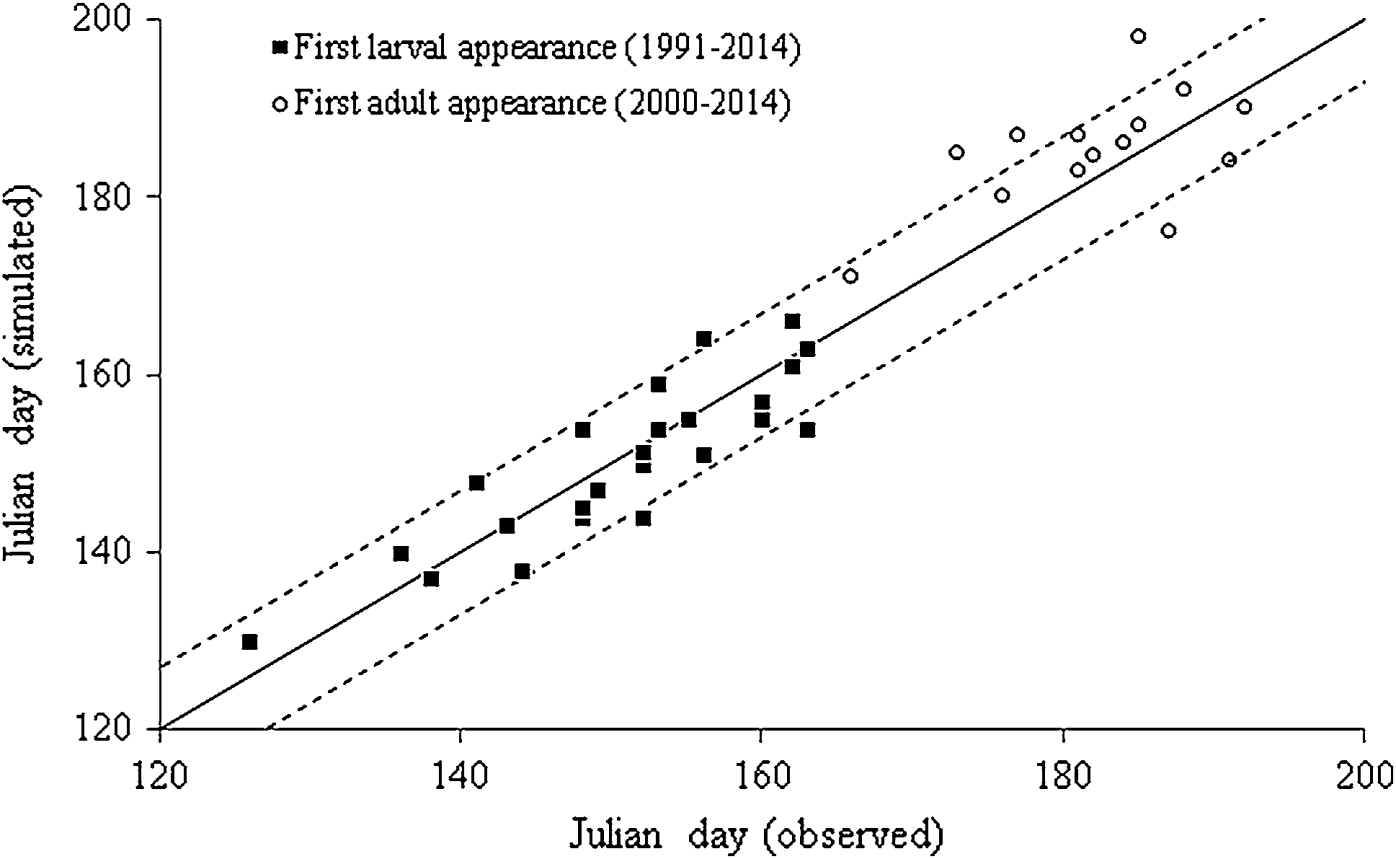
Observed versus simulated first larval and adult appearance in Central Illinois 1991–2014. The *solid line* represents the 1:1 *line* and the *dashed lines* represent a variation of ±1 week

Differences between measured and simulated behaviours within each season were much smaller. The first appearance of larvae and adult emergence was simulated for most years with a difference to field observations of <1 week (Fig. 2), whilst over- and underestimations for larval appearance balance each other out over the years we compared. Data on adult emergence are less frequently simulated within an error of 1 week, and simulations show a tendency towards an under-prediction of development (i.e. emergence is simulated later than actually occurred). Despite these uncertainties, the large variability of planting, damage assessment timing, larval and adult emergence and the large assumption of equal pest pressure across the years, the model simulates NIS well when considering the 10 years as a whole.

**Fig. 3:**
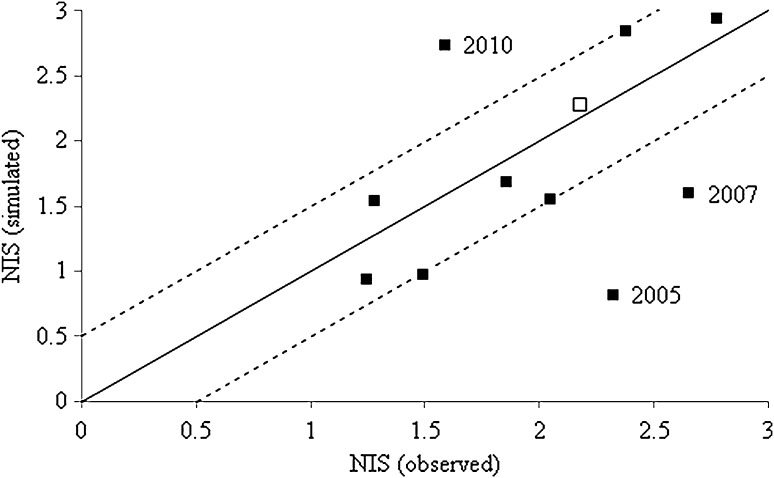
Observed versus simulated node injury in central Illinois 2005–2014. The *solid line* represents the 1:1 *line* and the *dashed lines* represent a variation of ±0.5 on the node injury scale. The *white* data point represents the NIS for Monticello 2014

Figure 3 presents the comparison between observed and simulated node injury from the control plots of the efficacy studies conducted in Urbana between 2005 and 2014 and from the field study in Monticello 2014. All simulations were conducted using the input parameters given in the methodology for model evaluation, the egg density of the Monticello field study (137 eggs/7.6 L soil) under tillage, and the actual planting and injury assessment days for the studies found in the literature (Table OR5). Overall, in eight out of the 11 years evaluated (73 %), the simulated node injury was within 0.5 points of that observed. The model over-predicted the root damage in 2010 and under-predicted root damage for the years 2005 and 2007 based on the assumption that pest pressure was the same as that measured in Monticello in 2014.

## Discussion

Temperature-dependent prediction of egg hatch and adult emergence is not a new development and has been undertaken for several decades. The new feature in the POPP-Corn model regarding the temporal prediction of rootworm populations is that rootworm development is not simulated according to a normally distributed developmental rate over all individuals, but rather developmental rate is fixed and stochasticity derives only from variations in soil temperature across the soil profile. This change in approach does not impact on the accuracy of simulating population development in terms of larvae and adult appearance even though there is a tendency of under-prediction of adult appearance (Fig. 2). The latter tendency should be interpreted with caution because some of the observations are accompanied with an uncertainty of roughly one week. The newsletter where these data are stated is published with one issue per week during the growing season; where actual dates for observed rootworm emergence were not stated explicitly, the date of the newsletter reporting that emergence had begun was used as a proxy. Furthermore, it is known that male adults start emerging approximately four days earlier than females (Meinke et al. 2009) and we used average developmental rates for all life stages in our model rather than modelling the two sexes separately.

Whether the over- and underestimation of node injury in 2005, 2007 and 2010 (Fig. 3) derives from pest pressure in those years that deviated from that at Monticello in 2014 cannot be verified, because actual egg densities were not determined during the trials (personal communication, Professor Michael E. Gray, Department of Crop Sciences, University of Illinois) or at locations nearby. This lack of determination of egg density as a measurement of pest pressure is a common feature of the literature base for research into root damage due to corn rootworm. The influence of egg density on predicted NIS is significant and can make the difference between a useful and a poor prediction (Figure OR4). We cannot tell whether variable egg density in the years analysed in Figure OR4 or extreme weather conditions at a crucial time for rootworm and root system development were the sole driver for the mismatch of observed and simulated NIS, nor how much such events have contributed to the discrepancies found. In depth analysis of the simulated temperature and water content data at sowing depth for the years analysed in Figure OR4 suggests that the underestimation of NIS in 2005 and 2007 might have been caused by inaccuracy of the root growth sub-model under drought. Root growth in POPP-Corn is, apart from root emergence, not dependent on water content in the soil. Central Illinois was reported to have been dry over the growing season in both years (Water and Atmospheric Resources Monitoring Program, Illinois Climate Network 2015) and the average water content at sowing depth between planting date and first rootworm adult emergence in 2005 and 2007 was found to be 24 and 23 % lower, respectively, compared to 2014 (which was used as input for pest pressure). It is thus possible that an overestimation of root growth may have contributed to the discrepancies found. Water content does not seem to have been the driving factor for the overestimation of NIS in 2010 because there were no large differences between weather data in 2010 and 2014 which would generate such an effect. Neither the average water content nor the average temperature at sowing depth between planning and adult emergence varied significantly between 2010 and 2014. Thus, it is more likely for 2010 that the pest pressure of 137 eggs/7.6L of soil (2014 value) was higher than that present in the field. Reduced egg density could have resulted from a very wet spring and subsequent increased egg mortality. It is known that the natural mortality of eggs is related to flooding (Stevo and Cagan 2010) and that survival of larvae is reduced in saturated soil (Hoback et al. 2002).

Assumptions about egg placement in the POPP-Corn model could be a further driving factor of discrepancies between observed and simulated node injury. For the model, it is assumed that row spacing and row placement are uniform between seasons for the purpose of egg placement in the soil profile. This assumption is particularly important to allow egg placement for a model run without conventional tillage where there is little mechanical redistribution of eggs within the field and placement at hatch relies strongly on adult egg-laying behaviour. We did not investigate this further as all simulations under the egg distribution pattern following tillage resulted in a good agreement between simulated NIS and that observed in the field.

One limitation is that this model has only been tested for fields with high pest pressure where pest capture crops were purposely planted in the year preceding the field study to ensure intermediate to high root damage ratings. Unfortunately, we could not test how well the model performs at intermediate and low pest pressures as knowledge on the initial pest pressure is vital for such fields but currently, to our knowledge, not recorded. Adult emergence rates from the preceding year could potentially be used to calculate the number of eggs in the field. This strategy is, however, accompanied with a high uncertainty in egg numbers as the number of eggs laid per female can vary greatly depending on the overall condition of females. Egg numbers per female have been reported to vary between average and standard deviations of 266 ± 133 and 1087 ± 217 (Spencer et al. 2009).

We have not explored how well POPP-Corn is able to predict differences in root damage from corn rootworm feeding at different locations of pest infestation within the USA and in other affected countries. Yet, the ability of the model to cope with the fluctuating climatic conditions over the years tested together with the ability to adjust the temperature threshold for egg development inspires confidence that POPP-Corn is applicable to other regions. There might, however, be a need to enhance the model with a formal description of the impact of water content in the soil profile on survival of eggs and larvae.

The development of POPP-Corn was undertaken using only ecological information for the western corn rootworm *Diabrotica virgifera virgifera* and model evaluation has also only been conducted on information for this species; nevertheless, we assume that the model could be readily adapted for all *Diabrotica* species due to their very similar life histories.

Future extensions for the presented model which interlink population development with, for example, toxicity of agrochemicals and Bt corn could be a vital development to be able to analyse the season- and region-independent efficacy of pest control measures. The latest attempt to evaluate season- and region-independent efficacy of pest control measures (Tinsley et al. 2015) was able to show differences in the efficacy by chemical control and use of Bt corn and other control measures. They generated linear regressions which help in understanding the relative efficacy of options that are available to manage corn rootworm injury, but their analysis is accompanied by large variabilities in the NIS because control measures were aggregated and did not take account of differences in weather, pest development, planting date or assessment date. An extension of our model might be able to separate the various control measures further and so add value to the analysis of relative efficacy.

## Electronic supplementary material

This manuscript includes one Online Resource (OR) consisting of five Tables, four Figures, a description of the feeding assay and additional details for the temperature and water content modelling.

## Author contribution statement

CB, PS and RA conceived the research. CB modelled the temperature and water content profiles. Experimental data for Monticello 2014 and experimental facilities were supplied by Syngenta and arranged by PS. All authors were involved in the model design. AA conducted the feeding experiment, wrote the model code, conducted the model testing and drafted the manuscript. AA, CB and RA finalised the manuscript. All authors read and approved the manuscript.

## Electronic supplementary material

Below is the link to the electronic supplementary material.

Supplementary material 1 (DOCX 314 kb)
