# Supplementary material for: Prediction of pest pressure on corn root nodes: the POPP-Corn model
Source: J Pest Sci (2004). 2016 Jun 21;90(1):161–72. doi: 10.1007/s10340-016-0788-x (PMC5290061; doi:10.1007/s10340-016-0788-x)
Supplement: Supplementary file 1 — Supplementary material 1 (DOCX 314 kb) [file 10340_2016_788_MOESM1_ESM.docx]

**Prediction Of Pest Pressure on Corn Root Nodes – The POPP-Corn model**

Annika Agatz ^a^ *, Roman Ashauer ^a^, Paul Sweeney ^b^, Colin D. Brown ^a^

^a^ Environment Department, University of York, Heslington, York, United Kingdom

^b^ Syngenta, Jealott's Hill, Bracknell, United Kingdom

* Corresponding author : [annika.agatz@york.ac.uk](mailto:annika.agatz@york.ac.uk) + 44-(0)-1904323118

**Journal of Pest Science - Online Resource**

**Number of Tables: 5**

**Number of Figures 4**

Table OR1: Parameters in the POPP-Corn model.

| **Procedure** | **Parameter** | **Description** | **Value** | **Unit** |
| --- | --- | --- | --- | --- |
| Root growth | *rg* | Root mass increase per root section | 0.000914 | [g/d] |
|  | *pru* | Increase in pruning by every eaten and pruned root segment | 0.00004 | [ ] |
| Oviposition | *ed1* | Relative egg density in soil layer 0-10 cm | 21 | [%] |
|  | *ed2* | Relative egg density in soil layer 11-20 cm | 45 | [%] |
|  | *ed3* | Relative egg density in soil layer 21-30 cm | 24 | [%] |
| Larval movement | *mf_sand_* | Soil dependent movement frequency for sand | 24 | [/d] |
|  | *mf_silt_* | Soil dependent movement frequency for silt loam | 6 | [/d] |
|  | *mf_sandy_* | Soil dependent movement frequency for sandy loam | 4 | [/d] |
|  | *l1m* | Larval instar 1 specific maximum root density for attractiveness | 0.00094 | [g] |
|  | *l2m* | Larval instar 2 specific maximum root density for attractiveness | 0.02 | [g] |
|  | *l3m* | Larval instar 3 specific maximum root density for attractiveness | 0.05 | [g] |
|  | *sd* | “Sensing distance” maximum distance within which a larvae can identify roots as food source | 5 | [cm] |
| Pest development | *de* | Developmental rate for eggs per degree above Bte | 0.0002 | [ 1/(h *°C)] |
|  | *dl1* | Developmental rate for larvae 1 per degree above Btl1 | 0.0006523 | [ 1/(h *°C)] |
|  | *dl2* | Developmental rate for larvae 2 per degree above Btl2 | 0.00058069 | [ 1/(h *°C)] |
|  | *dl3* | Developmental rate for larvae 3 per degree above Btl3 | 0.00027449 | [ 1/(h *°C)] |
|  | *dp* | Developmental rate for pupa per degree above Btp | 0.0003437 | [ 1/(h *°C)] |
| Pest survival | *me* | Mortality probability for eggs per year (normally distributed (average and standard deviation)) | 0.184 ± 0.0104 | [ ] |
|  | Data on larval establishment success from the literature were fitted with a Sigmoidal Hill function with three parameters as proxy for survival of larval instars 1. | | | |
|  | *ml1a* | First parameter for natural mortality of larval instar 1 | 0.9684 | [ ] |
|  | *ml1b* | Second parameter for natural mortality of larval instar 1 | -6.3041 | [ ] |
|  | *ml1c* | Third parameter for natural mortality of larval instar | 59.0668 | [ ] |
|  | *ml2* | Mortality probability for larval instar 2 per year (normally distributed (average and standard deviation)) | 0.4053 ± 0.0319 | [ ] |
|  | *ml3* | Mortality probability for larval instar 3 and pupa together per year (normally distributed (average and standard deviation)) | 0.36 ± 0.0321 | [ ] |
| Larval feeding | *frl1* | Feeding rate of larvae 1 | 0.000015 | [g/h] |
|  | *frl2* | Feeding rate of larvae 2 | 0.000034 | [g/h] |
|  | *frl3* | Feeding rate of larvae 3 | 0.000063 | [g/h] |

Table OR2: Variables for the randomised normal distribution (mean ± standard deviation) of root segments across the horizontal and vertical places of the soil profile in the POPP-Corn model.

| **Variable / Parameter** | **Description** | **Value/Equation** | **Unit** |
| --- | --- | --- | --- |
| hrrd1 | Maximum distance of root segment belonging to the root node 1 from the horizontal centre | 0 ± 2 | [cm] |
| hrrd2 | Maximum distance of root segment belonging to the root node 2 from the horizontal centre | 0 ± 5 | [cm] |
| hrrd3 | Maximum distance of root segment belonging to the root node 3 from the horizontal centre | 0 ± 8 | [cm] |
| hrrd4 | Maximum distance of root segment belonging to the root node 4 from the horizontal centre | 0 ± 15 | [cm] |
| hrrd5 | Maximum distance of root segment belonging to the root node 5 from the horizontal centre | 0 ± 19 | [cm] |
| hrrd6 | Maximum distance of root segment belonging to the root node 6 from the horizontal centre | 0 ± 22 | [cm] |
| hrrd7 | Maximum distance of root segment belonging to the root node 7 from the horizontal centre | 0 ± 30 | [cm] |
| vrrd1 | Maximum distance of root segment belonging to the root node 1 from the soil surface downwards | 0 ± vrrdf1*ageN1 | [cm] |
| vrrd2 | Maximum distance of root segment belonging to the root node 2 from the soil surface downwards | 0 ± vrrdf1*ageN2 | [cm] |
| vrrd3 | Maximum distance of root segment belonging to the root node 3 from the soil surface downwards | 0 ± vrrdf3*ageN3 | [cm] |
| vrrd4 | Maximum distance of root segment belonging to the root node 4 from the soil surface downwards | 0 ± vrrdf3*ageN4 | [cm] |
| vrrd5 | Maximum distance of root segment belonging to the root node 5 from the soil surface downwards | 0 ± vrrdf5*ageN5 | [cm] |
| vrrd6 | Maximum distance of root segment belonging to the root node 6 from the soil surface downwards | 0 ± vrrdf6*ageN6 | [cm] |
| vrrd7 | Maximum distance of root segment belonging to the root node 7 from the soil surface downwards | 0 ± vrrdf7*ageN7 | [cm] |
| *vrrdf1* | Factor for maximum distance of a root segment belonging to the root node 1 from the soil surface downwards per day of root node 1and 2 existence | 1.6 | [cm/d] |
| *vrrdf3* | Factor for maximum distance of a root segment belonging to the root node 3 from the soil surface downwards per day of root node 3 and 4 existence | 0.8 | [cm/d] |
| *vrrdf5* | Factor for maximum distance of a root segment belonging to the root node 5 from the soil surface downwards per day of root node 5 existence | 0.75 | [cm/d] |
| *vrrdf6* | Factor for maximum distance of a root segment belonging to the root node 6 from the soil surface downwards per day of root node 6 existence | 0.7 | [cm/d] |
| *vrrdf7* | Factor for maximum distance of a root segment belonging to the root node 7 from the soil surface downwards per day of root node 7 existence | 0.12 | [cm/d] |

Table OR3: Fixed and flexible variables within the POPP-Corn model.

| **Variable** | **Unit** | **Description** | **Value/Equation** |
| --- | --- | --- | --- |
| ageN1 | [d] | Age of the root node 1 | For t_j_>N1d  ageN1(t_j_) = ageN1(t_j-1_) + 1 |
| ageN2 | [d] | Age of the root node 2 | For t_j_>N2d  ageN2(t_j_) = ageN2(t_j-1_) + 1 |
| ageN3 | [d] | Age of the root node 3 | For t_j_>N3d  ageN3(t_j_) = ageN3(t_j-1_) + 1 |
| ageN4 | [d] | Age of the root node 4 | For t_j_>N4d  ageN4(t_j_) = ageN4(t_j-1_) + 1 |
| ageN5 | [d] | Age of the root node 5 | For t_j_>N5d  ageN5(t_j_) = ageN5(t_j-1_) + 1 |
| ageN6 | [d] | Age of the root node 6 | For t_j_>N6d  ageN6(t_j_) = ageN6(t_j-1_) + 1 |
| ageN7 | [d] | Age of the root node 7 | For t_j_>N7d  ageN7(t_j_) = ageN7(t_j-1_) + 1 |
| Bte | [°C] | Baseline temperature for egg development | Input variable (11°C for central Illinois and central Indiana) |
| Btl1 | [°C] | Baseline temperature for larval instar 1 development | 10.15 |
| Btl2 | [°C] | Baseline temperature for larval instar 2 development | 7.55 |
| Btl3 | [°C] | Baseline temperature for larval instar 3 development | 8.05 |
| Btp | [°C] | Baseline temperature for pupa development | 9.7 |
| DevE | [ ] | Development for eggs | DevE(t_j=0_) = 0  For DevE(t_j-1_) < 1and ST_i_(t_j_) ≤ 24  DevE(t_j_) = DevE(t_j-1_) + (de * (ST_i_(t_j_) – Bte)  For DevE(t_j-1_) < 1and ST_i_(t_j_) > 24  DevE(t_j_) = DevE(t_j-1_) + ((de * (ST_i_(t_j_) – Bte) – (24 – Bte)) |
| DevL1 | [ ] | Development for larval instar 1 | DevL1(t_j=0_) = 0  For DevL1(t_j-1_) <1  DevL1(t_j_) = (DevL1(t_j-1_) + (dl1 * (ST_i_(t_j_) – Btl1)) * Starv_i_(t_j_) |
| DevL2 | [ ] | Development for larval instar 2 | DevL2(t_j=0_) = 0  For DevL2(t_j-1_) <1  DevL2(t_j_) = (DevL2(t_j-1_) + (dl2 * (ST_i_(t_j_) – Btl2)) * Starv_i_(t_j_) |
| DevL3 | [ ] | Development for larval instar 3 | DevL3(t_j=0_) = 0  For DevL3(t_j-1_) <1  DevL3(t_j_) = (DevL3(t_j-1_) + (dl3 * (ST_i_(t_j_) – Btl3)) * Starv_i_(t_j_) |
| DevP | [ ] | Development for pupa | DevP(t_j=0_) = 0  For DevP(t_j-1_) <1  DevP(t_j_) = DevP(t_-1_) + (dp * (ST_i_(t_j_) – Btp) |
| Dlf1 | [g] | Direct feeding of all larvae from instar 1 | Dlf1(t_j_) = Σ Dlf1_i_(t_j_)  Dlf1_i_(t_j_) = frl1 * Starv_i_(t_j_) |
| Dlf2 | [g] | Direct feeding of all larvae from instar 2 | Dlf2(t_j_) = Σ Dlf2_i_(t_j_)  Dlf2_i_(t_j_) = frl2 * Starv_i_(t_j_) |
| Dlf3 | [g] | Direct feeding of all larvae from instar 3 | Dlf3(t_j_) = Σ Dlf3_i_(t_j_)  Dlf3_i_(t_j_) = frl3 * Starv_i_(t_j_) |
| Estab | [ ] | Establishment success of larval instar 1 (see SL1 for details) | Estab = 0 for freshly hatched larvae  Estab = 1 for individuals of larval instar 1 having reached a patch with a root segment available |

Table OR3 continued

| **Variable** | **Unit** | **Description** | **Value/Equation** |
| --- | --- | --- | --- |
| Hend | [cm] | Normal distribution for horizontal egg placement under absence of tillage | 7 ± 10 (Mean ± SD) form the left and right border of the soil profile towards the centre |
| L1a | [h] | Larval instar 1 age since hatch | L1a(t_j=0_) = 0  L1a(t_j_) = L1a(t_j-1_) + 1 |
| Lf_i_ | [g] | Total larval feeding within one patch | Lf_i_(t_j_) = Nl1_i_(t_j_) * frl1 + Nl2_i_(t_j_) * frl2 + Nl3_i_(t_j_) * frl3 |
| N1d | [d] | Days after root emergence for start of node 1 development | 0 |
| N2d | [d] | Days after root emergence for start of node 2 development | 8 |
| N3d | [d] | Days after root emergence for start of node 3 development | 16 |
| N4d | [d] | Days after root emergence for start of node 4 development | 24 |
| N5d | [d] | Days after root emergence for start of node 5 development | 32 |
| N6d | [d] | Days after root emergence for start of node 6 development | 40 |
| N7d | [d] | Days after root emergence for start of node 7 development | 48 |
| Ndr | [ ] | Number of dead root segments | Ndr(t_j=0_) = 0  Ndr(t_j_) = Ndr(t_j-1_) * ΣNdr_i_(t_j_) |
| Ndr_i_ | [ ] | Number of dead root segments per patch and day | - |
| Ne | [ ] | Number of eggs (autumn count) within the upper 30 cm of the soil profile | Input variable |
| Nl1_i_ | [ ] | Number of individuals in larval instar 1 within one patch | - |
| Nl2_i_ | [ ] | Number of individuals in larval instar 2 within one patch | - |
| Nl3_i_ | [ ] | Number of individuals in larval instar 3 within one patch | - |
| Nrs | [ ] | Number of root segments | - |
| PRU | [ ] | Pruning (survival probability of root segments) | PRU(t_j_) = PRU(t_j-1_) + (Ndr(t_j_) * *pru*) |
| Rd_i_ | [g] | Root density of a root segment within one grid | Rd_i_(t_j_) = Rd_i_(t_j-1_) + (*rg* * (1 – (PRU(t_j_)/100))) – Lf_i_(t_j_) |
| Rd_max_ | [d] | Age of maximum root density increase for a root segment | 30 |
| Rm | [g] | Root mass of the whole root system | Rm(t_j_) = ΣRd_i_(t_j_) |
| Sl1 | [ ] | Survival probability of larval instar 1 | Sl1(t_j_) = 1 – (*ml1a* * L1a(t_j_) ^ *ml1b* /( *ml1c* ^ *ml1b* + L1a(t_j_) ^ *ml1b*) |
| Sl2 | [ ] | Survival probability of larval instar 2 | Sl2(t_j_) = 1 – (*ml2* / ( 1 / DevL2(t_j_))) |
| Sl3 | [ ] | Survival probability of larval instar 3 | Sl3(t_j_) = 1 – (*ml3* / ( 1 / DevL3(t_j_))) |
| ST_i_ | [°C] | Soil temperature within a one cm horizontal soil profile zone | Input variable |
| Starv_i_ | [ ] | Starvation variable within each patch | For Nrs_i_(t_j_) > ΣNl _i_(t_j_)  Starv_i_(t_j_) = 1  For Nrs_i_(t_j_) < ΣNl _i_(t_j_)  Starv_i_(t_j_) = Nrs_i_(t_j_) / ΣNl _i_(t_j_) |
| TLf | [g] | Total larval feeding consisting of feeding from all larvae and pruning as consequence of direct feeding | TLf(t_j_) = Dlf1(t_j_) + Dlf2(t_j_) + Dlf3(t_j_) + PRU(t_j_) |

Table OR4: Details for the soil-temperature dependent development of all soil based life stages within the POPP-Corn model.

|  | **Egg** | **Larval instar 1** | **Larval instar 2** | **Larval instar 3** | **Pupa** |
| --- | --- | --- | --- | --- | --- |
| Baseline temperature [°C] | Flexible | 10.15 | 7.55 | 8.05 | 9.7 |
| Temperature of maximum development [°C] | Baseline temp.  + 24 | 30 | 30 | 30 | 30 |
| Development beyond temperature of maximum development | YES | NO | NO | NO | NO |
| Hourly relative development / local °C within temperature boundary | 0.0002 | 0.00065 | 0.00058 | 0.00027 | 0.00034 |

Table OR5: Summary of the literature data used for evaluation of the POPP-Corn model.

| Year | Observed  egg hatch  (Julian day)  (1) | Observed  adult emergence  (Julian day)  (2) | Planting day (Julian day)  (3)(4) | Damage assessment day  (days after planting) (3) | Observed  NIS  (5) | Number of treatments in the field averaged  (6)(7) |
| --- | --- | --- | --- | --- | --- | --- |
| 2014 | 153 | 181 | 132 | 72 | 1.25 | 4 |
| 2013 | 155 | 184 | 136 | 63 | 1.49 | 3 |
| 2012 | 126 | 166 | 109 | 82 | 2.05 | 4 |
| 2011 | 156 | - | 131 | 61 | 1.28 | 4 |
| 2010 | 149 | 176 | 124 | 69 | 1.58 | 5 |
| 2009 | 152 | 191 | 108 | 95 | 2.38 | 3 |
| 2008 | 156 | 192 | 115 | 89 | 1.85 | 5 |
| 2007 | 138 | 187 | 121 | 69 | 2.65 | 3 |
| 2006 | 152 | 181 | 118 | 80 | 2.77 | 3 |
| 2005 | 148 | 182 | 123 | 71 | 2.32 | 1 |
| 2004 | 141 | 177 | - | - | - |  |
| 2003 | 148 | 185 | - | - | - |  |
| 2002 | 153 | 185 | - | - | - |  |
| 2001 | 136 | 173 | - | - | - |  |
| 2000 | 143 | 188 | - | - | - |  |
| 1999 | 152 | - | - | - | - |  |
| 1998 | 148 | - | - | - | - |  |
| 1997 | 162 | - | - | - | - |  |
| 1996 | 163 | - | - | - | - |  |
| 1995 | 160 | - | - | - | - |  |
| 1994 | 162 | - | - | - | - |  |
| 1993 | 160 | - | - | - | - |  |
| 1992 | 163 | - | - | - | - |  |
| 1991 | 144 | - | - | - | - |  |
|  |  |  |  |  |  |  |
| (1) <https://extension.entm.purdue.edu/pestcrop/pastyears.html>  (2) Day at which rootworm emergence was mentioned in the weekly "Pest & Crop" Newsletter  (3) Day at which seeds where placed in the soil and day after planting at which plants were removed from the soil for damage assessment.(from the Annual Summary of Field Crop Insect Management Trials “On Target” from the University of Illinois Extension and Department of Crop Sciences)  (4) Row spacing 30 inches = 76.2 cm  (5) Average of all "Untreated checks (UTCs)" from the Annual Summary of Field Crop Insect Management Trials “On Target” from the University of Illinois Extension and Department of Crop Sciences  (6) Number of treatments within the field study used as "Untreated checks (UTCs)"  (7) Mean node-injury ratings from the field derived from five root systems checked per treatment in each of four replications (=20 plants checked per treatment) | | | | | | |


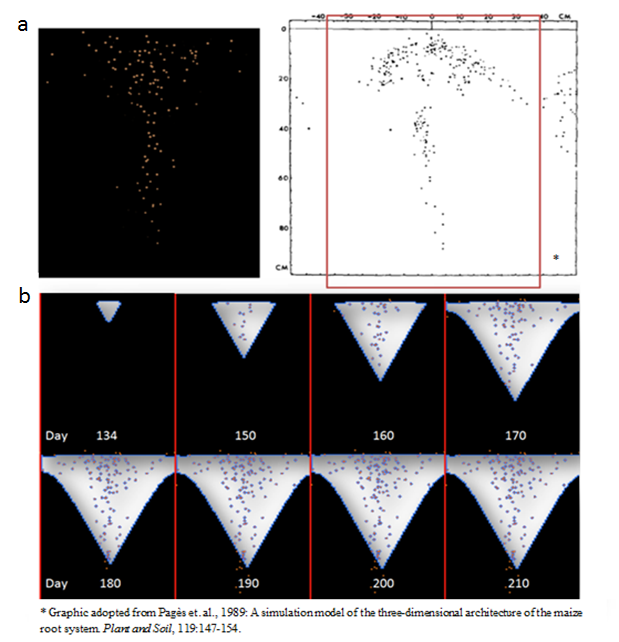


Figure OR1: Comparison of spatially- and temporally- explicit representation of corn root development between the root-growth sub-model of the POPP-Corn model and a) a 2D representation of a 3D root developmental model 50 days after planting, and b) a 2D root model (Daisy, which is a modification of the soil plant system model from Hansen 1993) over time (Julian day).

Dots represent spatially-explicit positioning of root segments from the POPP-Corn and the 3D root development model. White triangles represent the area where root mass is available within the Daisy model.


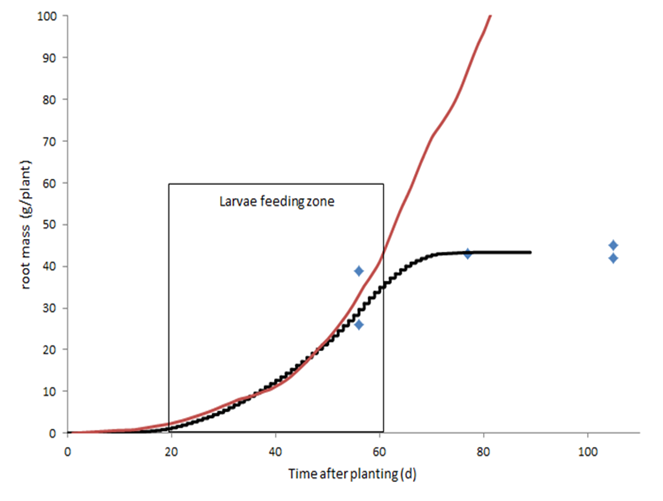


Figure OR2: Comparison between empirical data and model simulations for root mass production of a corn root system over time. Shown are empirical data by Anderson 1988 (blue diamonds), the POPP-Corn simulation (black line) and the simulation according to a two-dimensional soil plant system model from Hansen 1993 (red line).


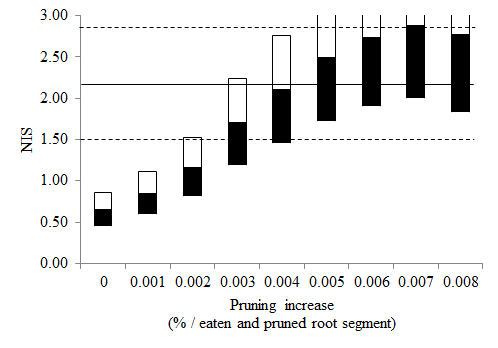


Figure OR3: Node injury for Monticello, IL 2014 simulated as a function of the value for the input parameter describing root pruning [*pru*]. Shown are the average (boundary between white and black boxes) and the upper (white) and lower (black) 95% confidence intervals simulated, and the average (solid line) and the 95% confidence intervals (dashed lines) for observed node injury.

The value for the root pruning parameter [pru] that resulted in a simulated NIS comparable with that observed in Monticello 2014 (i.e., similar average and 95% confidence interval) was used for all model runs conducted for this article.


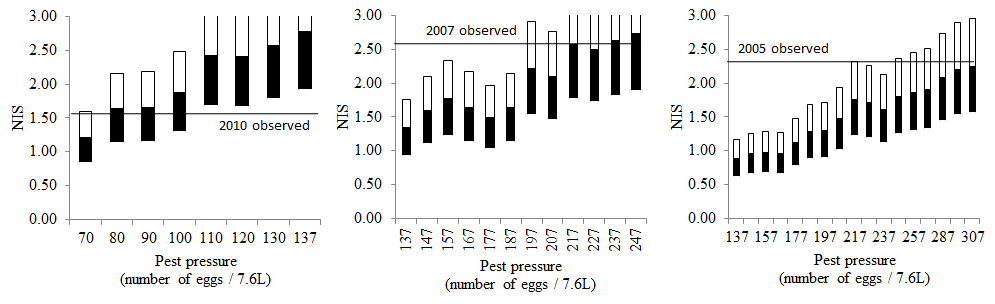


Figure OR4: Node injury for Urbana, IL 2010, 2007 and 2005 simulated as a function of the pest pressure. Shown are the average (boundary between white and black boxes) and the upper (white) and lower (black) 95% confidence intervals simulated, and the average (solid line) for observed node injury.

**Input parameters used for the temperature profile and water content modelling in MACRO**

Environmental input data for the PoPP-Corn model were generated using soil characteristics of the clay loam (32, 47 and 21% clay, silt and sand, respectively) from the study site in Monticello, IL 2014 with a pH of 5.5, an organic matter content of 3.5% and a cation exchange capacity of 20.3 meq/100g (Unpublished data, Syngenta). Weather data used as driving variables were taken from Bondville, Central Illinois, USA (Water and Atmospheric Resources Monitoring Program, Illinois Climate Network 2015) and comprised daily values for precipation, maximum and minimum air temperature, wind speed, relative humidity and solar radiation.

**Feeding assay**

Aim: Measuring the feeding rate of the three larval instars of the corn rootworm *Diabritica balteata* on fresh corn roots using gravimetric analysis.

Method: Whatman filter paper with a diameter of 1.5 cm was placed into 24 wells of a 24 well plate and dampened with 0.5 ml tap water each. Fresh root material with a known mass between 20.2 and 27.7 mg each was placed onto the wet filter paper. Root material derived from 4 day old corn seedlings were cut to 1.0-1.5 cm long root segments. Six rootworm larvae of each instar were placed individually into one well, and six wells were left larval free to be used as control. Larvae of the instars 1-3 had a head capsule width of 0.223 ± 0.047 mm, 0.335 ± 0.053 mm and 0.583 ± 0.068 mm, respectively. The well plate was placed into a growth chamber in total darkness at a temperature of 20 ± 1°C for a feeding period of 18.75 hours. Following the feeding period, larvae were removed from the wells and the remaining root material was transferred into a fresh 24 well plate. Remaining root material was left to dry for two days at room temperature and subsequently, its mass was determined. The control treatment was used to determine the ratio between wet and dry weight of the root material.

Results: The rootworm larvae of instars 1to 3 were observed to have eaten 0.0146 ± 0.0174 mg, 0.0343 ± 0.0106 mg and 0.0629 ± 0.0174 mg of root material (dry weight), respectively per hour and larvae.
